# Sub-Saharan African women’s views and experiences of risk factors for obstetric fistula: a qualitative systematic review

**DOI:** 10.1186/s12884-022-05013-2

**Published:** 2022-09-03

**Authors:** Lydia Babatunde Bulndi, Deborah Ireson, Esther Adama, Sara Bayes

**Affiliations:** 1 Edith Cowan University, 270 Joondalup Drive, Joondalup, WA 6027 Australia; 2 The Centre Evidence Informed Nursing, Midwifery and Health Care Practice; 270 Joondalup Drive, Joondalup, WA: A JBI Affiliated Group, Joondalup, Australia; 3 School of Nursing, Midwifery, and Paramedicine (Melbourne), Australian Catholic University, 115 Victoria Parade Fitzroy, Victoria Melbourne, 3065 Australia

**Keywords:** Birth injury, Birth experiences, Obstetric fistula, Risk factors, Sub-Saharan Africa, Vesico Vaginal Fistula, Women’s view

## Abstract

**Background:**

Obstetric fistula used as synonymous with VVF in this study, is an abnormal communication/hole between the urinary tract and the genital tract or the gastrointestinal tract and the genital tract, resulting from prolonged obstructed labour. VVF may cause sufferers to experience chronic urinary/faecal incontinence, and the stigma of continuing foul odour. VVF is primarily caused by prolonged obstructed labour, which is brought about by a range of causes. Recently, it has been proposed that women’s groups and fistula survivors should suggest interventions to reduce or prevent the incidence of obstetric fistula.

**Objective:**

The objective of this review was to synthesise what is reported about women’s views and experiences of the risk factors underlying the causes of VVF.

**Methods:**

A systematic approach outlined in the Joanna Briggs Institute Manual for Evidence synthesis was followed for this review, articles published since the last 11 years from 2011 to 2021 were selected against several criteria and critically appraised using JBI Critical Appraisal Checklist for qualitative studies.

**Results:**

Nine studies were retained for inclusion in this review and the data were then synthesised into five themes: (1) Cultural beliefs and practices impeding safe childbirth, (2) Lack of woman’s autonomy in choices of place to birth safely, (3) Lack of accessibility and social support to safe childbirth, (4) Inexperienced birth attendants and, (5) Delayed emergency maternal care (childbirth).

**Conclusions:**

This review highlights the complexity of risk factors predisposing women to the known causes of VVF. It also illuminates the absence of women’s voices in the identification of solutions to these risks. Women are most directly affected by VVF. Therefore, their knowledge, views, and experiences should be considered in the development and implementation of strategies to address the issue. Exploring women’s views on this issue would enable the identification of gaps in maternity care provision, which would be of interest to community and health service leaders as well as policymakers in Sub-Saharan Africa.

**Supplementary Information:**

The online version contains supplementary material available at 10.1186/s12884-022-05013-2.

## Background

Obstetric fistula is 'an abnormal communication/hole between the urinary tract and the genital tract or the gastrointestinal tract and the genital tract, resulting from prolonged obstructed labour [1, 2]. There are many types of obstetric fistulas including vesicovaginal fistula (VVF) which occurs between the bladder and vagina, rectovaginal fistula (RVF) which occurs between the rectum and the vagina, ureterovaginal fistula (UVF), between the urethra and vagina, ureterovaginal fistula, between the ureters and the vagina. In this study, VVF will be used as being synonymous with obstetric fistula. VVF may cause sufferers to experience chronic urinary/faecal incontinence, the stigma of continuing foul odour, social exclusion, and decreased quality of life [3–5]. VVF is primarily caused by prolonged obstructed labour, for which there are several known contributing factors such as giving birth at home in the absence of a midwife or skilled birth attendant can increase the likelihood of VVF [6].

According to the World Health Organisation [7], each year between 50,000 to 100,000 women worldwide are affected by VVF. The majority of women affected by VVF are from low-income countries such as Sub-Saharan Africa with under-resourced ineffective health care systems [3]. In developed high-income countries, VVF are rare and usually occur from bladder injury during gynaecologic procedures or radiation therapy [8]. The disparity in incidence between high and low-income countries is likely due to poor availability of midwifery and skilled birth attendants [9, 10]. In low- and middle-income areas such as Sub-Saharan Africa, VVF remains a public health concern [11–13] because of its far-reaching effects on women.

VVF is caused by several factors such as poor maternal care, lack of access to skilled care during labour, physical factors, and economic incapability to access care during labour [14, 15]. Most women with VVF in developing countries often start labour at home without a midwife’s supervision which can lead to prolonged, obstructed labour and subsequent development of VVF [16]. Additionally, early marriage and teenage pregnancy expose young women to complicated labour leading to VVF formation [17, 18]. Other contributing factors of VVF include lack of emergency transportation to hospitals and competent healthcare workers at the facility [19, 20]. When a delayed decision to take the woman to a hospital is made or there is lack of autonomy for the woman to go to the hospital without the consent of her husband, the woman’s access to skilled birth services is either delayed or in some instances outrightly hindered by either spouse or family members [16, 21]. A woman with no form of education may not understand the importance of antenatal care attendance nor the need for hospital birth. Birthing practices at home are influenced by cultural and religious practices; these practices can contribute to VVF development [19, 22].

The consequences of VVF have a devastating ongoing impact on the lives of the women, families, and communities [3, 23, 24]. The most immediate effect of VVF is that the woman experiences either single or double incontinence, leading to social stigma and social isolation; it also has negative emotional health impacts such as psychological trauma and depression [25]. There is a similarly devastating high rate of stillbirth among women who develop VVF, although the estimates of cases in which the two co-occur are variable and imprecise [26]. Notwithstanding, the association between VVF and stillbirth is clear, with most sufferers of obstetric fistula reporting a stillbirth [1, 24]. The result for many women affected by VVF is that their key social roles such as motherhood, wife or daughter are lost because they are unlikely to have a living child and are often abandoned by their husbands and wider family network [3, 27]. Diminished capacity to socialise because of incontinence and its associated odour also occurs in women affected by VVF, which leads to decreased employability and financial hardship [3], resulting in erosion of self-worth and psychological trauma [28–30]. Lastly, recurrent fistula is also a common complication of pregnancy among women previously affected by VVF, even after surgical repair of earlier injury [31, 32].

The multidimensional consequences of VVF require an understanding of women’s experiences of risk factors to guide the antenatal preparation of women for labour/birth and intrapartum management of women [3]. More research that involves primary health initiatives, where grassroot women’s groups and fistula survivors suggest interventions that will reduce or prevent the incidence of VVF in their communities has been advocated [25]. To begin to address this call, this review was conducted to identify and synthesise recent qualitative studies on Sub-Saharan African women’s views and experiences of risk factors associated with VVF occurrences and to determine any knowledge gap in the available literature on this topic. Understanding Sub-Saharan women’s experiences of VVF is an important step in designing interventions that meet their unique needs and experiences.

### Review question

The specific review question was “What is the most recent qualitative evidence on Sub-Saharan African women’s views and experiences of risk factors associated with obstetric fistula occurrences?”. Reviewing current evidence will enhance healthcare workers’ understanding of current trends and experiences of women living with VVF.

## Methods

### Design

The qualitative systematic review followed the process for conducting systematic reviews as outlined in the Joanna Briggs Institute (JBI)manual for writing a qualitative systematic review as described by [33]. The stages are as follows:(a) developing a search strategy, (b) reviewing the literature and (c)developing data extraction techniques and data synthesis. Population, Phenomenon of Interest, Context, study design (PICOS) framework was used to facilitate the development of the review question, search terms, and inclusion and exclusion criteria [27, 28] (see the Logic Grid in Table 1). A priori systematic review protocol was developed and agreed upon by all authors before commencing the review. All disagreements on inclusion or exclusion were resolved through discussions. The Preferred Reporting Items for Systematic review and Meta-Analyses(PRISMA)checklist [34] criteria were adhered to for reporting this review.

**Table 1:** Logic Grid: “What are the qualitative evidence on women’s views and experiences of risk factors associated with obstetric fistula occurrences in Sub-Saharan African?”

Population	Phenomenon of Interest	Context	Study Design
Women	Perceptions of risk factors of obstetric fistula Experiences of obstetric fistula Views of affected women and girls regarding vesico- vaginal fistula Perspectives on recto-vesico-vaginal fistula risks	Sub Saharan Africa	Qualitative Mixed method (qualitative data included only)

### Search strategy

Four electronic databases (CINAHL, PubMed, Web of Science, and Google Scholar) were searched to find recent articles (published since 2011). Qualitative studies or qualitative components of mixed-methods studies were sought. The initial database search was performed in September 2020, and a verification search was completed on2^nd^March 2021. Keywords and NIH National Library of Medicine Medical Subjects’ Heading (MeSH) [35] terms were combined for searching (Table 2). Boolean terms “AND” and “OR” were applied where appropriate to focus the search as much as possible (see Table 2). The reference lists of the retrieved articles were then hand-searched to identify any additional studies that met the inclusion criteria.

**Table 2:** Final search terms

The search terms included: ("women’s perception" [MeSH Terms]) OR ("women’s view [MesH Terms]) OR ‘’ ("women’s experiences" [MeSH Terms]) OR (" Women’s health"[MeSH Terms]) OR ("women’s perspectives" ([MeSH Terms]) OR "Women’s perception’’[ MeSH Terms]) AND ("risk factors" [MeSH Terms]) AND ("obstetric fistula" [MeSH Terms]) OR ("vesico vagina fistula" [MeSH Terms]) OR ("recto vesico-vaginal fistula" [ MeSH Terms]) OR (‘'Urogenital-vaginal fistula’’ [ MeSH Terms]) AND ("Sub-Saharan African). "
.

### Search terms/keywords

The keywords “Obstetric fistula”, Vesicovaginal fistula”, “rectovaginal fistula”, “Urogenital-vaginal fistula’’ “Reproductive women’’ “Women View’’, “Women Perception’’, “Women’s experience’’, “Women’s health’’’Risk factors’’, were used to search relevant papers see Table 2., the search protocol was as follows:

Following the JBI manual for qualitative systematic reviews, three steps were used to select papers for review [33]. Firstly, relevant titles and abstracts were identified from databases. Secondly, screening and retrieving of full-text articles were conducted. In the final stage, after identifying papers that potentially meet the inclusion criteria, data extraction from relevant selected articles and quality reviews of the articles was done. Analysis was restricted to studies intended to explore the Sub-Saharan African women’s views and their experiences of risk factors for obstetric fistula.

### Inclusion and exclusion criteria

For studies that have an abstract identified in the database searches, the abstracts were reviewed to determine whether they should be included by using pre-defined inclusion and exclusion criteria. Articles included in the review were: (i) primary qualitative research or mixed-method study designs, (ii) studies that included women of reproductive age, (iii) studies focusing on women’s perspectives and experience of risk factors of obstetrics fistula, (iv) peer-reviewed studies published in English language (v) studies published between 2011–2021 and (vi) studies conducted only in Sub-Saharan Africa. Articles were excluded if they were: (i) Studies focusing solely on consequences of obstetric fistula, (ii) Studies outside sub-Sahara Africa, (iii) Studies focusing on awareness of risk factors of obstetric fistula, and (iv) Quantitative studies.

### Method of the review

Identified papers were read in full and included only if they reported women’s views and their experiences of risk factors for obstetric fistula. The standardised critical appraisal instrument from the JBI Qualitative Assessment and Review instrument (JBI-QARI) was used for each study included [33] (See Appendix II). Full text of included studies were assessed with the listed 10 criteria in JBI-QARI such as congruity between the stated philosophical perspective and the research methodology; congruity between the research methodology and the research objectives; congruity between the research methodology and the methods used to collect data; congruity between the research methodology and the representation and analysis of data; congruity between the research methodology and the interpretation of results; statement locating the researcher culturally or theoretically, influence of the researcher on the research, and vice-versa addressed, are participants and their voices, adequately represented, research ethical according to current criteria or, for recent studies, and is there evidence of ethical approval by the appropriate body, conclusions drawn in the research report flow from the analysis, or interpretation of the data. Each study had scored high in each criterion with a quality rating score of high to low for each article. Two authors independently assessed all included studies, and the third and fourth authors then verified the assessments. All authors then came together to reach consensus about which articles to include in the study. All the included studies were found to be of sound scientific quality.

### Data extraction

Qualitative data were extracted from included articles using the standardised data extraction tool from JBI-QARI [33] (See Appendix III).The data extracted included specific details about the author, title, country, Study design /methodology, sample size/participant, aim of the study and findings (Table 3).

**Table 3:** Characteristics of Nine Qualitative/ Mixed Methods Studies Reporting Risk Factors of Obstetric Fistula in Sub Saharan Africa

No	Authors	Title	Country	Study design /Methodology	Sample size/Participant	Aim of the study	Findings
1	Ahmed et al., 2020 [16]	Childbirth experiences of Sudanese women living with obstetric fistula	Sudan	Qualitative/ Thematic Analysis	19 women, semi -structured interview	The study seeks to provide a better understanding of the circumstances surrounding the occurrence of obstetric fistula	Family members did not allow the women to go to hospital for 3 days. Failure of the birth attendant to recognise danger signs at home
2	Bangser., 2011 [6]	Childbirth experiences of women with obstetric fistula in Tanzania and Uganda and their implications for fistula program development	Tanzania and Uganda	Mixed method/ including Participatory approach	Semi-structured interviews, 137 women Quantitative survey	Study explores whether women's experiences of their “near-miss” deaths and experiences living with fistula could provide essential information for strengthening maternal health policies and programs and those specifically addressing fistula	Participant testimonies expand current understanding of women's experience of fistula
3	Boene et al., 2020 [27]	Obstetric fistula in southern Mozambique: a qualitative study on women’s experiences of care during pregnancy, delivery, and post-partum Norther Nigeria	Mozambique	Qualitative /phenomenological approach	14 Women in-depth interviews	Describes women’s experiences of antenatal, intrapartum, and postpartum care in southern Mozambique, to pinpoint those experiences that are unique to women with fistula to understand the care-seeking and care provision circumstances which could have been modified to avoid or mitigate the onset or consequences of fistula	Deficiencies and delays in birth assistance, referral and life-saving interventions were commonly reported by women with fistula
4	Changole et al., 2018 [36]	A road to obstetric fistula in Malawi: capturing women’s perspectives through a framework of three delays	Malawi	Qualitative study/ Social constructivism perspective and interactionism	25 women semi -structure interview	To understand labour and delivery experiences of women who develop obstetric fistula in Malawi	Finding shows decisions to seek health care when labour is complicated were made by mothers-in-law and traditional birth attendance
5	Degge et al., 2020 [14, 15]	Insights from birthing experiences of fistula survivors in north-central Nigeria: Interplay of structural violence	Nigeria	Qualitative study/Narrative inquiry	15 women, Narrative inquiry	This study examines the social structures surrounding the formation of obstetric fistula among women	The study evidenced obstetric fistula, as a product of structural violence occurring in a country with poor health system
6	Kaplan et al., 2017 [37]	An investigation of the relationship between autonomy, childbirth practices, and obstetric fistula among women in rural Lilongwe district, Malawi	Malawi	Qualitative study/ Grounded theory	25 women, in depth qualitative interview	This study assessed whether women’s limited autonomy in rural Malawi reinforces childbearing practices that increase risk of obstetric fistula	Study showed women are required to seek permission from husbands to visit the antenatally clinic and labour ward
7	Mwini- Nyaledzigbor et al., 2013 [2]	Lived experiences of Ghanaian women with obstetric fistula	Ghana	Qualitative study/Descriptive approach	10 women, semi structured interviews	Explores the experiences of Ghanaian women who sustained obstetric fistula during childbirth	Study shows the combination of poor status, lack of education, and poor access to healthcare facility led to fistula
8	Mselle et al., 2011 [38, 39]	Waiting for attention and care: birthing accounts of women in rural Tanzania who developed obstetric fistula as an outcome of labour	Tanzania	Qualitative and Quantitative Study/Survey and interpretative approach	16 women semi-structured interview, 151 quantitative surveys	To use both qualitative and quantitative to explore the birthing experiences of women affected by obstetric fistula, and barriers to accessing adequate quality of care during labour and birth	Study reveals a series of weaknesses in the health care system associated with obstetric competence, infrastructure, and health worker -women relationship
9	Mselle et al., 2015 [40]	Perceived health care system causes of obstetric fistula from accounts of affected women in rural Tanzania a qualitative study	Tanzania	Qualitative study /Thematic Analysis	16 women semi -structured interviews, 12 focus groups	The study explored and described perceived health system causes of obstetric fistula from affected women	Women’s perceptions emphasize the importance of improving the quality of obstetric care provided by health care facilities

### Data synthesis

Qualitative research findings were pooled using JBI-QARI which involved the aggregation or synthesis of findings to generate a set of statements [33]. These stages are (I) coding of identified excerpts from the selected studies (ii) developing descriptive themes from the codes by aggregating similar ideas (iii) developing analytical themes (See Table 4). The first author analysed all data, and authors 2–4 each analysed a selection of data; all data were analysed by two people independently. All authors then came together to reach a consensus about the final themes.

**Table 4:** Descriptive and analytical themes

Descriptive Theme	Analytical Theme
Cultural beliefs and practices of traditional birth attendants (TBA)	Cultural beliefs and practices impeding safe childbirth
The inability of women to make decisions related to safe childbirth	Lack of woman’s autonomy in choices of place to birth safely
Lack of transportation, financial hardship, and absence of social support	Lack of accessibility and social support for safe childbirth
Poorly skilled attendants	Inexperienced skilled birth attendant
Poorly assisted health facility childbirth (Vacuum or Forceps)	Delayed emergency maternal care (childbirth)

## Results

### Search result

**Fig. 1:**
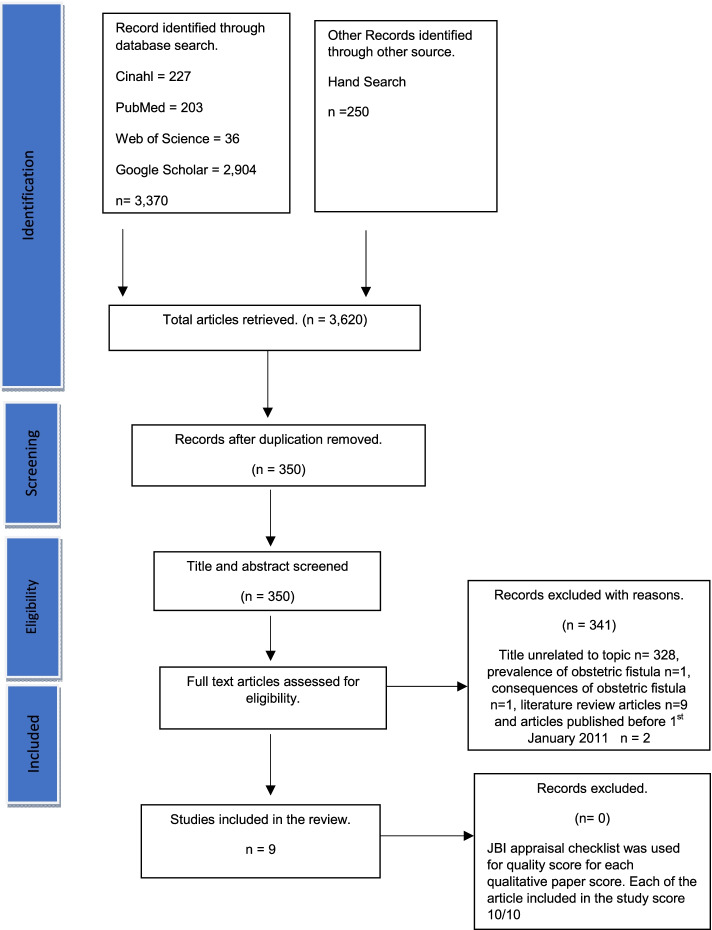
PRISMA flow diagram

A total of 3,620 articles were retrieved. After removing duplicates, 350 articles were retained for screening. However, 341 of the articles were further excluded for reasons such as being solely focused on the prevalence of VVF, consequences of VVF, or because they were literature review articles, unrelated titles, and/or published before January 2011. Overall, nine articles met the inclusion criteria. Of the nine articles, seven were qualitative studies and two were mixed methods research. The search result is shown in PRISMA flow diagram (Fig. 1). All included studies were conducted in Sub-Saharan Africa: one in Ghana [2], one in Nigeria [15], three in Tanzania and Uganda [6, 38, 40], two in Malawi [36, 37], one in Mozambique [27], one in Sudan [16]. All the studies were conducted in a treatment or rehabilitation facility. The methodological quality of the included articles scored high in each criterion with a quality rating score of high to low for each article. The thematic categories shared across most studies were related to cultural beliefs and practices impeding safe childbirth; lack of women’s autonomy in choices of place to birth safely; lack of accessibility and social support for safe childbirth and inexperienced birth attendant and delayed emergency maternal care (childbirth).

### Theme 1: Cultural beliefs and practices impeding safe childbirth

Three articles cited cultural beliefs and practices as a risk factor expressed by women as a cause of VVF [1, 2, 16]. These articles reported cultural beliefs and practices impeded safe childbirth reporting outcomes including VVF and foetal death. Some of the cultural beliefs and practices included unskilled birth attendants such as matriarchal figures and traditional untrained birth attendants rather than qualified midwives. First-time mothers or primiparous women were asked by relatives to wait at home and birth with the help of unskilled birth attendants who have little or no physiological understanding of labour and birth. Other cultural practices include asking women to confess an adulterous act to pacify the gods for safe birthing. One participant related to this in the following quotation:

*“I was in labor from night to the following day evening and I was in pain and tired, but instead of taking me to the hospital, they rather suspected that I had committed adultery and that was why the labour was difficult. The old women [TBA] insisted that I confess to enable the baby to come out. But I also insisted on my innocence. Not convinced by that, they went and had some consultations with their gods and offered some sacrifices” *[2]

### Theme 2: Lack of women’s autonomy in choices of place to birth safely

Women’s lack of negotiating power and ready acceptance of others’ decisions were reported in four studies [2, 16, 37, 39]. This theme shows that women are not consulted in decision-making concerning the choice of healthcare or place of birth instead, a male guardian, traditional birth attendant, husband, or father takes patriarchal authority. For example, one woman discussed this factor in the development of VVF as follows:

*“I was in labour pains for three days at home with a traditional birth attendant. I didn’t think about going to the hospital since my family would not allow me to go to the hospital because they say women who deliver[birth] for the first time should wait and be patient”*[16]*.*

### Theme 3: Lack of accessibility and social support for safe childbirth

Findings from seven studies highlighted this theme [1, 6, 16, 36, 37, 39, 40] in which women expressed difficulty in accessing health care facilities because of distance, poor roads, lack of finances, and lack of suitable transportation (including delays in hire vehicles and/or lack of finances to hire a vehicle). For instance, one woman described her experience as follows*:*

*“The hospital is far, therefore, it took some time to find the money to rent a car to take me there, it took us a whole day to reach the hospital, on reaching the hospital, they put me up on the delivery table, but they told me that the baby had died’’ *[16]*.*

Poverty impeded access to healthcare and planning for the care needed when women are in labour as expressed by a woman who related her VVF to

“I plan to deliver[birth] at the hospital but it was not possible because I did not have cash and I did not know where I will get money from, it was January, and during the farming season, I did not go to the field, my husband went, I did not go, and it was then that I got these problems’’ [39]

Lack of social support from (friends or family) to convey the parturient woman to a health facility was also reported:

“Labour pains started suddenly around midnight, and I started for the hospital. But since I was all alone and walking slowly struggling along the way due to labour pains, it took time. But if only I had someone, to take me on a bicycle, maybe I would have gotten to the hospital in time. So, while I was on the way to the hospital, my legs got cold; were numb, and I could no longer walk. So, I thought of just sitting down. Then I just saw that legs have started coming out. I said ‘’ah! …ah! what is this ?’’ I tried to stand, but I could not manage. So just remained seated, all alone. So, I just remained seated, all alone. So when the thing [ baby] finally stretched its legs and came out, I saw that the thing was already dead [Nangozo, 13 years living with fistula] [36]

### Theme 4: Inexperienced birth attendant

Aggregation of findings from three studies made up this theme [1, 16, 36]. Women’s previous encounters with inexperienced and neglectful healthcare providers during childbirth were cited as an indirect risk factor for VVF. Women perceived young skilled birth attendants as inexperienced in providing quality obstetric care as compared to mature TBAs. This potentially inaccurate perception of skilled birth attendants and the TBA’s lack of knowledge and skills in managing obstructed labour can result in the development of VVF. One woman recalled:

“At the hospital nowadays there are many small children [young midwives] who attend to you, so you think to yourself “ah, it is better to go to that old woman to help me, moreover, at the hospital, they are not even there to receive your baby when it is coming, you do the work [push] by yourself and call them when the baby is out, while at the Azamba [TBA], she is always there’’ [36]

These perceptions about skilled birth attendants have led many women to seek care from traditional birth attendants who lack knowledge in recognising obstructed labour (Changole et al. 2018).

### Theme 5: Delayed emergency maternal care (childbirth)

This theme was obtained from the analysis of findings from three articles [27, 39, 40]. This theme shows that delays in receiving emergency care on reaching a health care facility constitute a risk for the development of VVF among women of childbearing age. Lack of essential emergency services associated with a shortage of staff or equipment, including delay in referring women to health care facilities with available emergency services was highlighted. As an example, one woman related the development of her VVF to the following scenario*:*

“I was in labour for a long time while in the hospital, labour pain comes and goes and each time when you call, nurses tell you, wait, though I had very

strong pains. I didn’t see the reason why I could not be sent for operation early…I think nurses contributed to my problem’’. [40]

## Discussion

This study synthesised the qualitative findings of nine studies to explore Sub-Saharan African women’s views and experiences of risk factors for obstetric fistula as evidence for intervention studies and for the progression of policy aimed at reducing the incidence of VVF. There is a paucity of review on this subject. Moreover, as the trend in healthcare delivery changes rapidly, the women's views and experiences could shape the strategic policies around healthcare delivery in line with inclusiveness and equity of healthcare provision globally.Five analytical themes were identified: ‘Cultural beliefs and practices impeding safe childbirth, lack of woman’s autonomy to choices of place to birth safely, lack of accessibility and social support to safe childbirth, inexperienced birth attendants and delayed emergency maternal care services in childbirth; all these themes represent what is currently known about what women’s views are and their experiences of risk factors underlying the causes of VVF in Sub-Saharan African.

Our findings highlighted how cultural beliefs and practices impede women from seeking skilled childbirth attendants. The fact that some women do give birth successfully in the context of such beliefs at home with a traditional birth attendant (TBA) has reinforced this approach in the community; however, the focus on spiritual and magical drivers of labour and birth put reproductive women at risk of VVF and/or stillbirth [2]. As [36] asserted, TBAs’ lack of understanding of birth physiology and the nature, meaning, and impact of prolonged or obstructed labour is crucial in the development of VVF [36]. The result is that women are kept at home for an extended period before referral to the hospital; in some cases, they are never referred to the hospital even when complications are evident [41]. However, a contrast to this pattern was reported in the Ethiopian health care system, where TBAs’ role was acknowledged as that of a volunteer worker, attending to women under the supervision of health extension workers; although the role and relationship have not been clearly defined [42]. However, programs that incorporate a participatory approach such as that outlined by [42] seem to offer a model of care for women in labour where TBAs are given a well-defined roles such as birth companions or interpreters for women in labour [43]. Midwives currently working in communities can be a useful resource for training TBAs to a competent level, if there role are well-defined this will improve health care services to meet the needs of rural women and family needs [43]. However, it is essential that the provision of childbirth care, preferences, and needs, including having a companion of choice in labour be considered by midwives and other skilled birth attendants to reduce the risk of obstetric fistula in the community [43–45].

Our review also elucidates limited decision-making power on the part of women regarding where to give birth. This choice is primarily made by the woman’s husband, mother, the TBAs, her mother-in-law, grandmothers, or other relations instead of the women [2, 16, 37, 39]. The data in the articles we reviewed concurs with a study undertaken at Sokoto Northern Nigeria [46] in which it was found that low patronage of modern maternal health facilities by women in labour was associated with limitations placed on women’s freedom to choose the health care centre as a place to birth safely. In this study, women could not access healthcare services without the permission of their husbands [46]. In Malawi, women’s lack of access to financial resources is implicated in this issue as this leads directly to women having limited autonomy on health care utilisation during labour and birth, which in turn increases their risk of developing obstetrics fistula [37]. In Northern Nigeria, the factors underlying the lack of decision-making power are similar, it is related to the practice of ‘’Purdah”, which involves wife seclusion whereby women are not allowed to go out to earn a living [25].

We also found issues of lack of accessibility and social support for safe childbirth environments, as few women have either an available means of transportation or support from husbands and relations in accompanying them to health institutions when they are in active labour, or the ability to afford medical supplies required by hospitals [1, 16, 27, 36]. Transportation is costly and unaffordable, or it is non-existent [20, 41, 47]. This finding is in line with that of [15], who described the lack of transportation for women in labour as structural violence against women [48] also implicated cost as a barrier to a safe birthing environment, and another reason why women seek traditional birth attendants’ services rather than birth in a health facility, is because women are asked to provide medical supplies or consumables that they cannot afford; in contrast, TBAs require nothing but a white piece of cloth. Pregnancy and childbirth issues in African countries are perceived to be ‘’women’s issues’’, and most men are culturally excluded from participating in maternal care or in accompanying their partner to the clinics [36, 49]. However, [49] study mirrored that, even though men are culturally excluded in maternal care, some men still want to be involved in healthcare issues of their spouses at critical times such as decision making, but were given little or no attention by the midwives at the clinic, therefore some men thought it to be time-wasting accompanying their spouse in labour to the clinic.

Another factor we identified in women developing VVF in this review, is women’s reluctance to attend dedicated maternity care facilities secondary to a lack of trust in those facilities’ caregivers and poor management experience encountered at places of birth [50, 51]. Women’s choice of place to birth was shaped by their negative past experiences with healthcare facilities during childbirth and their recall of healthcare providers as incompetent in handling their labour [20, 36, 42, 52, 53,54] suggest that a shortage of skilled experienced staff in the maternity units, or limited professional development might be reasons for skilled birth attendants’ limited exposure to (and therefore limited competence in managing) the array of complications they might be presented with, as well as increased workload or deteriorating staff morale. In contrast to our findings, [55] reported increased satisfaction of care received during childbirth among women in a dedicated maternity care facility. The researchers related the women’s satisfaction to the availability of qualified human resources including highly qualified midwives in the facility.

Finally, this review also highlighted delayed emergency maternal care services as an indirect cause of obstetric fistula. Lack of essential emergency services associated with a shortage of staff or equipment, including delay in referring women to a health care facility with emergency services such as emergency caesarean section was experienced at a healthcare facility [27, 39, 40]. This is in agreement with a study conducted in Uganda which report a lack of essential emergency obstetric services in the cases of an emergency including a lack of a functional referral system [52]. Women attribute lack of essential emergency services as an indirect cause of VVF including delay in referring women to a health care facility with emergency services.

### Strengths and limitations of the review

The objective of this review, which was to report what is currently known about “Sub-Saharan African women’s views and experiences of risk factors for obstetric fistula”, and to highlight gaps in knowledge about this topic, was fulfilled. One of the limitations is that the period of inclusion (2011–2021) may mean that earlier publication that might have yielded useful insights were not included; however, this period is essential in synthesising current evidence to deepen our understanding of the current trend of phenomenon under study. Secondly, the search was limited to articles published in the English language, and the omission of studies reported in other languages, particularly African languages, might have excluded useful data. Thirdly, grey literature, which may have provided additional insights into this topic, were excluded.

### Implications for practice and policy

- Future policies and initiatives should focus on culturally sensitive care which will incorporate a participatory approach for women in labour where TBAs will be given well-defined roles such as birth companions or interpreters for women in labour.
- There is a need to develop comprehensive strategies that are inclusive such as building more maternity hospitals to cater for women within the community to meet the needs of women in labour.
- Policy that encourages male involvement during labor are potential interventions to increase male involvement in pregnancy and childbirth issues.
- There is a need to train more midwives with the necessary skills required to prevent VVF through continuous midwifery programs.

## Conclusion

This review synthesised current qualitative findings of nine studies to understand Sub-Saharan African women’s views and experiences of risk factors for VVF. We found that cultural beliefs and practices impede safe childbirth; lack of woman’s autonomy in choices of place to birth safely; lack of accessibility and social support to safe childbirth, inexperienced birth attendant and delayed emergency maternal care services in childbirth together contribute to fistula formation. Although it is evident in the research published on this topic that women are well able to state what the factors underlying VVF are, it cannot be assumed that this review captures them all or that addressing these factors would mean a reduction in the incidence of obstetric fistula. To address the issue of VVF in Sub-Saharan Africa, further research is needed in the identification of solutions to these risks. Women are most directly affected by VVF. Therefore, their knowledge, views, and experiences should be considered in the development and implementation of strategies to address the issue. Research that is focused sharply on capturing all the issues that are implicated in VVF, and women’s proposed solutions to those issues, is warranted. Exploring women’s views on this issue would enable the development of remedial strategies, which would be of interest to community and health service leaders as well as to policy makers in Sub-Saharan Africa.

## Supplementary Information

**Additional file 1:** **Appendix I.** Search strategy. **Appendix II.** Appraisal instruments. **Appendix III.** Data extraction instruments. **Appendix IV.** Summary of thematic Analysis.

## Data Availability

Data sharing does not apply to this article as no datasets were generated or analysed during the current study.

## Abbreviations

VVF: Vesico Vaginal Fistula
JBI: Joanna Briggs Institute
PRISMA: Preferred Reporting Items for Systematic review and Meta-Analyses
PICOS: Population or Phenomenon of interest Context Study designs
TBAs: Traditional Birth Attendance(s)
