# Supplementary material for: Sub-Saharan African women’s views and experiences of risk factors for obstetric fistula: a qualitative systematic review
Source: BMC Pregnancy Childbirth. 2022 Sep 3;22:680. doi: 10.1186/s12884-022-05013-2 (PMC9440544; doi:10.1186/s12884-022-05013-2)
Supplement: Supplementary file 1 — Additional file 1: Appendix I. Search strategy. Appendix II. Appraisal instruments. Appendix III. Data extraction instruments. Appendix IV. Summary of thematic Analysis. [file 12884_2022_5013_MOESM1_ESM.docx]

# Appendix I: Search strategy

# *Record identified through database.*

# The initial database search was performed in September 2020,

# verification search was completed on 2^nd^March 2021

# Search thread

# *Cinahl*

# women’s perception" OR "women’s view OR "women’s experiences” OR “Women’s health" OR "women’s perspectives” OR "Women’s perception’’ AND "risk factors” AND Obstetric fistula OR Vesico vagina fistula” OR "Recto Vesico-vaginal fistula” OR 'Urogenital-vaginal fistula’’ AND "Sub-Saharan African. (227)

# *PubMed*

# women’s perception" OR "women’s view OR "women’s experiences” OR "Women’s health" OR "women’s perspectives” OR "Women’s perception’’ AND "risk factors” AND Obstetric fistula OR Vesico vagina fistula” OR "Recto Vesico-vaginal fistula” OR 'Urogenital-vaginal fistula’’ AND "Sub-Saharan African. (203)

# *Web of Science*

# women’s perception" OR "women’s view OR ‘’ women’s experiences” OR Women’s health" OR "women’s perspectives” OR "Women’s perception’’ AND "risk factors” AND Obstetric fistula OR Vesico vagina fistula” OR "Recto Vesico-vaginal fistula” OR 'Urogenital-vaginal fistula’’ AND "Sub-Saharan African. (36)

# *Google Scholar*

# women’s perception" OR "women’s view OR ‘’ women’s experiences” OR Women’s health" OR "women’s perspectives” OR "Women’s perception’’ AND "risk factors” AND Obstetric fistula OR Vesico vagina fistula” OR "Recto Vesico-vaginal fistula” OR 'Urogenital-vaginal fistula’’ AND "Sub-Saharan African. (2,904)

# *Other Records identified through other source.*

# Hand Search

# . A hand search by searching the reference list of included studies (250)

# Appendix II: Appraisal instruments

## QARI appraisal instrument

**JBI CRITICAL APPRAISAL CHECKLIST FOR QUALITATIVE RESEARCH**

**Reviewer**___________________________________**Date**____________________________

**Author**_______________________________ **Yea**r_________ **Record Number**_________

**Yes, No Unclear Not applicable**

1. Is there congruity between the stated philosophical **Yes No Unclear Not Applicable**

perspective and the research methodology? □ □ □□

2. Is there congruity between the research methodology

and the research question or objectives? □□□ □

3. Is there congruity between the research methodology

and the methods used to collect data? □ □ □ □

4. Is there congruity between the research methodology

and the representation and analysis of data? □ □ □ □

5. Is there congruity between the research methodology

and the interpretation of results? □ □ □ □

6. Is there a statement locating the researcher culturally

or theoretically? □ □ □ □

7. Is the influence of the researcher on the research, and

vice- versa, addressed? □ □ □ □

8. Are participants, and their voices, adequately

represented? □ □ □ □

9. Is the research ethical according to current criteria or,

for recent studies, and is there evidence of ethical

approval by an appropriate body? □ □ □ □

10. Do the conclusions drawn in the research report flow

from the analysis, or interpretation, of the data? □□ □□

Overall appraisal: Include □ Exclude □ Seek further info □

Comments (Including reason for exclusion)

**Appendix III: Data extraction instruments**

**
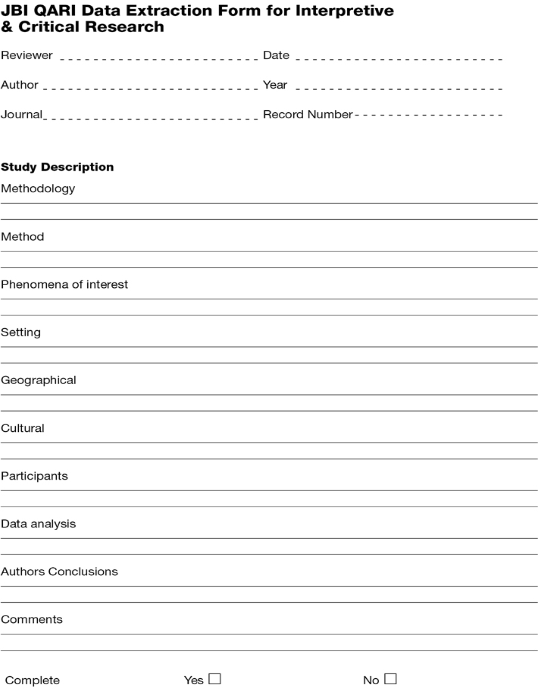
QARI data extraction instrument**

# Appendix IV: Summary of thematic Analysis

| Authors | Excerpts | Themes  (Analytical Themes) | Sub Themes  (Descriptive Themes) |
| --- | --- | --- | --- |
| Ahmed et al,  2020 | 1.“I was in labour pains for three days at home with a traditional birth attendance. I didn’t think about going to the hospital since my family would not allow me to go to the hospital because they say women who deliver for the first time should wait and be patient.”  2.The hospital is far. Therefore, it took some time to find the money to rent a car to take me there. It took us a whole day to reach the hospital. When I reached the hospital, they put me up at the delivery table, but they told me that this baby had died.  3. “When I had the pain [ labor pain] they brought me a midwife at home. The midwife started squeezing my tummy and telling me that I will deliver soon. I was feeling his movement until the night then it stopped.” I lost my child. | 1. Cultural beliefs and practices impeding safe childbirth.  1. Lack of woman’s autonomy in choices of place to birth safely.  1. Lack of Accessibility and social support for safe childbirth.  1.Inexperienced skilled birth attendant. | 1.Cultural beliefs and practices  1. Inability of women to make decision related to safe childbirth.  1.Lack of transportation, financial hardships, and absence of social support.  1.Poorly skilled attendants |
| Boene et al., 2020 | 1. I was assisted at [name of secondary-level hospital] ... [When] I arrived, they took a long time before performing my surgery … if they had operated on me in the same day that I arrived, maybe my baby would have lived because when I arrived there I was feeling the baby moving … “I first pushed, they [nurses] instructed me to push, so I pushed, but the baby was not coming out, something was blocking the front [the birth canal]. Then the nurses became worried and said “we don’t know what this is “Then they called the ambulance. Meanwhile, my mother’s [mother and mother-in-law] had gone to see a traditional healer and came back with a remedy that I drank and after that the baby was out …” | 1. Delayed emergency maternal care (childbirth) | 1.Poorly assisted health facility childbirth (Vacuum or Forceps). |
| Changole et al., 2018 | 1. [at the TBA], I overstayed without getting better, “a Zamba” [TBA] tried hard, touching me, pressing on the middle of my abdomen [presumably fundal pressure], to help the baby come out, but it did not. So I told her [TBA] to take me to the hospital, but she said, “No, do you think at the hospital they will just pull out the baby without you pushing? Will they not require you to do the same?” Then she [TBA] took a strip of reed and used it to stretch the opening, to widen it, but instead it was just cutting my flesh, and it failed. I was badly wounded. Then they (TBAs) … they were two of them, said, “Iih, we are cutting her”, so they stopped.Then some held my back, some held my mouth, and some held my abdomen. Then finally the baby dropped out; but dead. Meanwhile, I was badly injured. (Saying it with such feeling of betrayal). [Nadzimbiri, 12 years living with fistula]  2.Labor pains started suddenly around midnight, and I started off for the hospital. But since I was all alone and walking slowly struggling along the way due to labor pains, it took time. But if only I had someone, to take me on a bicycle, maybe I would have gotten to the hospital in time. So, while I was on the way to the hospital, my legs got cold; werenumb, I could no longer walk. So, I thought of just sitting down. Then I just saw that, legs have started coming out. I said “ah!..ah!, what is this?” I tried to stand, but I could not manage. So, I just remained seated, all alone. So, when the thing [baby] finally stretched its legs and came out, I saw that the thing was already dead. [Nangozo, 13 years living with fistula] | 2.Inexperienced skilled birth attendant.  2. Lack of accessibility and social supportto safe childbirth. | 2.Poorly attended home birth.  2.Lack of transportation, financial hardships, and absence of social support. |
| Degge et al., 2020 | 1.They decided to let them try the local way, ‘she will deliver’ … so they brought a freshly hatched chick and added this to a concoction so… that I will deliver right away, but no way. They put a big wooden stick [usedfor stirring ‘tuwo’- a local food] in my mouth and said that is how it is done. I will deliver right away… I could not urinate, and they even pounded and mixed a slimy vegetable and started pouring this into in my front side to force the head of the baby out. (Maryamu, 38yrs)  2. I couldn't take myself; ‘they’ were the ones to take me… but there was no money, so they couldn't take me to the hospital when labour started.  (Chungdung, 52yrs)  3. Since we are in the village [there is no hospital at that time], there was no car. So, they took me with the wheelbarrow to [city name provided], where the hospital in my state [province] is.  4. It was then a nurse in the hospital said she was going to sit on me here (touched her chest) to make the baby come out. I told her, “…you want to kill me”. She said it's because of my stubbornness that something bad will happen to me. The next thing I was looking at the machine, lying down… I heard the noise of a machine that they used to pull out the baby…grrrrrrrrrrrrrrrl… What I knew next was urine…and stool. (Maryamu, 38yrs) | 2. Cultural beliefs and practices impeding safe childbirth.  3. Lack of Accessibility and social support for safe childbirth.  4. Lack of Accessibility and social support for safe childbirth.  3. Inexperienced skilled birth attendant. | 2. Cultural beliefs and practices  3. Lack of transportation, financial hardships, and absence of social support.  4. Lack of transportation, financial hardships, and absence of social support.  3.Poorly attended health facility childbirth.  (Vacuum or Forceps)  Poorly home birth |
| Kaplan et al., 2017 | 1.There was “no way” we could leave without telling our husbands, and they described the underlying reason as the need to show respect and avoid problem.  2. My husband] told me that [if] I wanted to deliver at the hospital, I should find my own means of traveling there because he would not be able to support me.” | 3. Lack of women’s autonomy in choices of place to birth safely.  4. Lack of Accessibility and social support for safe childbirth. | 3. In ability of women to make decision related to safe childbirth.  4. Lack of transportation, financial hardships, and absence of social support. |
| Mwini-Nyaledzigbor et al., 2013 | 1. I was in labor from night to the following day evening and I was in pain and tired, but instead of taking me to the hospital, they rather suspected that I had committed adultery and that was why the labor was difficult. The old women [TBA] insisted that I confess to enable the baby to come out. But I also insisted on my innocence. Not convinced by that, they went and had some consultations with their gods and offered some sacrifices.  2. When I was in labor, my husband was not around. He had gone to another village the previous day to farm and considering the distance to the place nobody could reach him to inform him about my situation, neither could they take any decision in his absence. So, they waited for him to return, and that kept me in labor for 3 days. It was when he returned that the decision to take me to hospital was made. (Attaa) | 5. Lack of women autonomy in choices of place to birth safely.  3. Cultural beliefs and practices impeding safe childbirth.  6. Lack of women autonomy in choices of place to birth safely. | 5. In ability of women to make decision related to safe childbirth.  3.Cultural beliefs and practices  6. In ability of women to make decision related to safe childbirth. |
| Mselle et al., 2015  Mselle et al; 2015 | 1.(...) the day when I had labour pains, a nurse was not there (...) the baby's head was already out (...) I think if a nurse was around, I would not have ended with this problem, and if they would have failed, they could have taken me to theatre many hours earlier (...) (PM)  2.I was in labour for a long time while in the hospital. Labour pain comes and goes and each time when you call nurses tells you, wait, though I had very strong pains. I didn’t see the reason as to why I could not be sent for operation early...I think nurses contributed to my problem (FGD-BS).  3. If I was informed early about the problem, my family could take me to a big hospital for operation (...) (NM).  4. I think nurses contribute to the development of fistula. (…) they keep you waiting for so long in labour without making any decision of taking you to theatre for operation (FGD-AS) | 2.Delayed emergency maternal care childbirth.  3. Lack of Accessibility and social support for safe childbirth.  3. Delayed emergency childbirth services  4. Delayed emergency childbirth services | 5.Poorly attended health facility childbirth  3. Lack of transportation and financial hardship  5.Poorly attended health facility childbirth  5. Poorly attended health facility childbirth |
| Mselle et al., 2011 | 1.My parents said I should wait as I could deliver at home...you know in the village people do deliver in homes (...) in our village, there is a TBA, she is the one who harmed us” (35-year-old from Kisongo, in the dispensary, they did nothing; they did not check anything on me. I was left alone.... I was alone throughout the night, there was no doctor... they took me to Pande hospital in the morning, by then labour pain had ceased already” (35-year-old from KilwaKisongo).  2. From our village to Iseke (midway to Manyoni Hospital) is 24 kilometres, and from Iseke to Manyoni is very far, and there was no means of transport. Therefore, when labour pains started, everybody was confused because I could not walk, and there was no means of transport to take me to Manyoni Hospital. I therefore remained at home” (35-year-old from Singid  3.Labour pain started at home at around 1am. I stayed until at 3am when we found the pains were picking up, we took a taxi to dispensary. In the dispensary, I slept, spent the day, slept, and woke-up again, on the third day it was when a decision was made to transfer me to a big hospital. They said we are failing here because you have urine retention. That was when I was transferred to Tumbi hospital” (20-year-old from Mlandizi, Pwani). | 2. Lack of women autonomy in choices of place to birth safely.  5. Delayed emergency childbirth services  3. Lack of Accessibility to safe childbirth.  2.Lack of women autonomy in choices of place to birth safely.  6. Delayed emergency obstetric services | 4. In ability of women to make decision related to safe childbirth.  5.Poorly attended health facility childbirth  3. Lack of transportation and financial hardship  2. In ability of women to make decision related to safe childbirth.  5. Poorly attended health facility childbirth |
| Bangser., 2011 | 1.We moved on foot up to the bus stop, but it took us too long because I could only move a bit...Even before we left, my mother had to first sell a goat, and getting a willing buyer wasn't easy. Then when we got to the taxi stand, we had to wait one hour to get to the nearby health center” (Woman from Masaka, Uganda, 19) | 3. Lack of Accessibility to safe childbirth. | 3.Lack of transportation and financial hardship. |
